# Supplementary material for: Prognostic Value of Inflammatory and Nutritional Biomarkers of Immune Checkpoint Inhibitor Treatment for Recurrent or Metastatic Squamous Cell Carcinoma of the Head and Neck
Source: Cancers (Basel). 2023 Mar 28;15(7):2021. doi: 10.3390/cancers15072021 (PMC10093403; doi:10.3390/cancers15072021)
Supplement: Supplementary file 1 [file cancers-15-02021-s001.zip › cancers-2287638-supplementary.pdf]

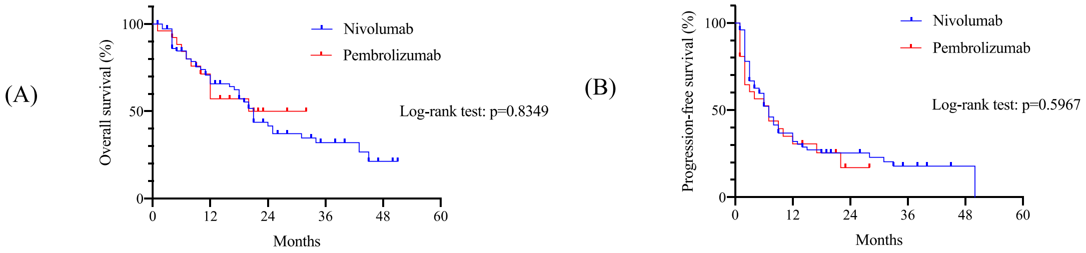

	Median (95%CI) OS (months)	1-year OS rate (%)	2-year OS rate (%)
Nivolumab	21 (17-25)	67.8	41.5
Pembrolizumab	20 (10-NA)	57.1	49.9

	Median (95%CI) PFS (months)	1-year PFS rate (%)	2-year PFS rate (%)
Nivolumab	7 (5-9)	32	25.4
Pembrolizumab	7 (2-12)	30.6	17

Figure S1. Kaplan–Meier curves of overall survival (**A**) and progression-free survival (**B**) according to the immune checkpoint inhibitors. Significant differences in OS and PFS were observed between Nivolumab and Pembrolizumab.
